# Removal of anthraquinone dye from wastewaters by hybrid modified activated carbons

**DOI:** 10.1007/s11356-023-27550-9

**Published:** 2023-05-17

**Authors:** Athanasia K. Tolkou, Athanasios C. Mitropoulos, George Z. Kyzas

**Affiliations:** Department of Chemistry, International Hellenic University, 65404 Kavala, Greece

**Keywords:** Remazol Brilliant Blue R, Anthraquinone dye, Activated carbon, Wastewater treatment

## Abstract

Dyes are among the main environmental pollutants, due to the high amount of discharge of wastewater, lost in the dyeing process, without any further treatment. Anthraquinone dyes are stable and resistant in the aquatic system. Among the methods that have been applied to remove these dyes from wastewaters, adsorption on activated carbon has been reported as a very effective technique, and its modification with oxides and hydroxides of metals have been used to increase its surface area. In the present study, the production of activated carbon was originated by coconut shells, and a mixture of metals and metalloids, such as magnesium, silicate, lanthanum, and aluminum (AC-Mg-Si-La-Al), was used for its subsequent modification and applied to Remazol Brilliant Blue R (RBBR) removal. AC-Mg-Si-La-Al surface morphology was studied by BET, FTIR, and SEM methods. For the evaluation of AC-Mg-Si-La-Al, several parameters, such as dosage, pH, contact time, and initial RBBR concentration were studied. According to the results, in pH 5.0 ± 0.1, the dye percentage rate reached 100% by applying 0.5 g/L. Therefore, the optimal dose of 0.4 g/L and pH 5.0 ± 0.1 are selected, which leads to 99% removal of RBBR. The experimental data found to better fit to Freundlich isotherm (*R*^2^ = 0.9189) and pseudo-second-order kinetic (*R*^2^ = 0.9291) models and 4 h were the sufficient time for adsorption. According to thermodynamics, a positive value of ∆H^0^ (19.661 kJ/mol) suggests the endothermic nature of the process. The AC-Mg-Si-La-Al adsorbent was able to regenerate after 5 cycles of use, showing only a 17% decrease in its efficiency. Because of its effectiveness in full RBBR removal, AC-Mg-Si-La-Al could be further examined for the removal of several other dyes, even anionic or cationic.

**Graphical Abstract:**

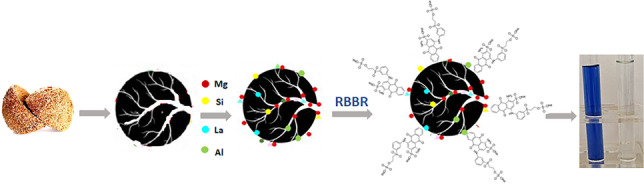

## Introduction

Environmental concerns related to wastewater effluents have become controversial among researchers worldwide and have forced scientists to look for simple, more economical, and immediate solutions for their treatment (Pal et al. 2022). Dyes are among the environmental pollutants due to their large amount deposited in wastewater (Farhan Hanafi and Sapawe 2020) and can be characterized as high-strength industrial wastewaters (Tolkou and Zouboulis 2020). Various types of dyes, used in industries (Yaseen and Scholz 2019), such as leather, food, cosmetic, paper, textile, and printing, are transferred to the effluents discharged from these industries (Arya et al. 2020a) without any further treatment, thus causing wide environmental pollution. Τhere are three categories of dyes used, such as non-ionic, cationic, and anionic, which are water-soluble and can be toxic even at low concentrations (Rahdar et al. 2021). As for instance, sugarcane bagasse pulp was recently used for the successful treatment of textile wastewater effluents including methylene blue dye (cationic) (Elshabrawy et al. 2023). Moreover, colorants are classified into natural and into the more commonly used synthetic dyes produced in various colors (Khehra et al. 2006). Synthetic dyes according to their chemical structure and different groups can be divided to azo, anthraquinone, sulfur, phthalocyanine, and triarylmethane and as per their applications can be described as reactive, direct, disperse, basic, and vat (Yeow et al. 2021). Most of these dyes are decomposed and provide high toxicity, carcinogenicity, and mutagenic products in water (Brüschweiler and Merlot 2017; Lellis et al. 2019).

Anthraquinone dyes have a serious impact on the environment as due to their complex structure is difficult to be naturally degraded (Routoula and Patwardhan 2020; Samchetshabam et al. 2017). The biggest environmental concern of the presence of dyes in water is related to the absorption and reflection of sunlight by them. Hence, light enters the water with the consequent reduction of the photosynthetic activity of the algae, which thus affects the food chain. A serious problem is also the fact that these dyes can remain in the environment for a long time, due to their high thermal stability (Samchetshabam et al. 2017).

**Fig. 1:**
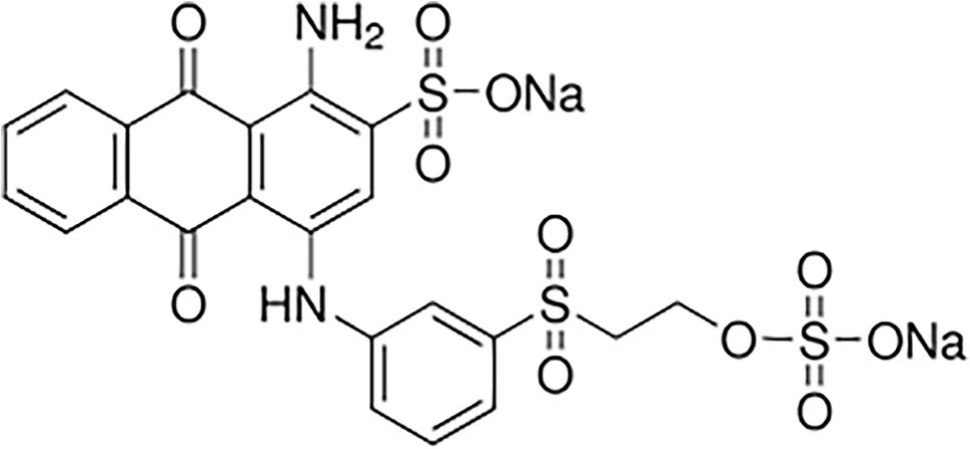
Structure formula of RBBR (Ahmad et al. 2020)

RBBR is a widely used anthraquinone dye (C_22_H_16_N_2_Na_2_O_11_S_3_; molecular weight: 626.54 g/mol) (Ahmad et al. 2020) (Fig. 1). It is soluble in water, making it a common contaminant found in industrial wastewaters, which is often used as a source substantial for the creation of polymeric colorants (Hadibarata and Kristanti 2012) and belongs to non-biodegradable, recalcitrant, and toxic organic pollutants (Silva et al. 2016).

Several methods, physical, biological, chemical and a combination of these methods, have been used for the elimination of dyes in wastewater (Al-Tohamy et al. 2022). However, studies found that some physical and chemical treatments do not completely inactivate dyes, which may cause secondary pollution when discharged into the environment. On the other hand, biological treatment is cost-effective, produces less sludge compared to physical and chemical treatments, but is less efficient for decolorization, depending on the microorganism used in treatment (Al-Tohamy et al. 2022; Piaskowski et al. 2018). The applied treatment includes precipitation (Faisal et al. 2022; Han et al. 2022; Li et al. 2022), oxidation (Peramune et al. 2022), coagulation (Ihaddaden et al. 2022; Mcyotto et al. 2021), adsorption (Al-Tohamy et al. 2022; Alhogbi et al. 2021; Farhan Hanafi and Sapawe 2020; Yaseen and Scholz 2019), and membrane separation (Raval et al. 2022; Zhao et al. 2023). Nevertheless, adsorption is a low-cost, simple, and a common method for the removal of organic dyes from wastewater (Gupta and Suhas 2009; Jia et al. 2017; Kandisa and Saibaba 2016; Kyzas et al. 2018; Ramakrishna and Viraraghavan 1997).

Several adsorbents, including zeolites (Senguttuvan et al. 2022; Zhang et al. 2022), alumina (Al-Salihi et al. 2022; Folawewo and Bala 2022), silica gel (Volikov et al. 2016), chitosan (Kyzas et al. 2014, 2013), magnetic nanocomposites (Rahdar et al. 2021), graphene oxide (Saroyan et al. 2019a, b; Travlou et al. 2013), and activated carbon (Azam et al. 2022; Gul et al. 2022; H. S. Saroyan et al. 2019a, b), have been widely used for dye removal from wastewater.

Activated carbon (AC), a porous carbonaceous substance, is one of the most effective and widely used adsorbents, which has been applied to wastewater treatment because of its operation, simplicity, and reusability (Vijayaraghavan et al. 2009). However, regardless of its wide application in wastewater treatment, certain commercial activated carbon remains an unprofitable material (Nizam et al. 2021). Consequently, researchers’ interest in lower-cost sources of activated carbon which maintain the advantages of a safe, effective, and economical dye removal method from wastewater has increased (Ramakrishna and Viraraghavan 1997). Therefore, activated carbons from different low-cost origin materials, such as agriculture waste and by-products (e.g., coconut shell and husks, bamboo, sugarcane, coffee waste, fruit peels, bagasse), have been produced in several studies (Devi et al. 2012; Gupta and Suhas 2009; Kyzas et al. 2022; Sarker et al. 2017; Tolkou et al. 2023, 2022). Therefore, there is need for the design and development of advanced and composite activated carbon materials that have high dye removal performance and efficiency.

Recently, many researchers have studied the modification of activated carbons with several additives, to increase its surface area, for their application in dye removal. Therefore, focusing on the additives used in this study, AC has been modified recently with aluminum (Al) (Kazeem et al. 2018) for cationic dye removal, but the dosage used was very high (5–10 g/L) and with lanthanum(III) (Goscianska et al. 2014) for the removal of methyl orange dye. Dye removal was also studied using modified silicate minerals (Selim et al. 2014) for methyl violet dye and polyvinyl pyrrolidone–modified magnesium oxide (Khan et al. 2022) for methyl orange and Congo red adsorption. In addition, recently, the authors of this study modified AC with a combination of magnesium, lanthanum, and silica for the efficient removal of fluoride anions from water (Tolkou et al. 2023).

The present study describes the application of activated carbon, produced from coconut shells, impregnated with a mixture of metals and metalloids, such as magnesium, silicate, lanthanum, and aluminum (AC-Mg-Si-La-Al), as an adsorbent to remove reactive anthraquinone dye RBBR. The aim of this research was the production of an adsorbent material, which would take advantage of the properties of each additional additive, but at the same time a low dose of it would be applied for the effective and complete removal of dye. To the best of our knowledge, to date, there is no study that has examined the use of such a modified material, i.e., the use of activated carbon derived from coconut shells modified with magnesium, silicate, lanthanum, and aluminum for dye removal. The effects of each additive present, the initial pH value, adsorbent’s dose, initial RBBR concentration, and contact time, were investigated. The structure and the morphology of the modified activated carbons were analyzed by BET, FTIR, and SEM/EDS. In addition, kinetic and isotherm models were used to interpret the adsorption process, and thermodynamics and regeneration studies were applied.

## Materials and methods

### Materials

#### Chemicals

RBBR dye used as an adsorbate and supplied by Sigma-Aldrich. A 1000-mg/L stock solution of RBBR dye (1.0 g diluted in 1000 mL deionized water) was produced and used for the preparation of required concentrations. MgO (PMS2 pure magnesia), SiO_2_ (Merck), LaCl_3_·7H_2_O 98% (Merck), and AlCl_3_·6H_2_O (Merck) were used for activated carbon’s modification. For pH adjustment, 0.01–0.1 M of HCl 37% (Panreac) or NaOH ACS reagent, ≥ 97.0%, and pellets (Sigma-Aldrich) were used. Moreover, 1.0 M NaOH was used for the regeneration study and NaCl (Sigma-Aldrich) solutions (0.1–1.0 M) for testing the effect of ionic strength on dye removal.

#### Synthesis of modified activated carbon

Activated carbon used in this research, as adsorbent, was produced initially by using coconut shells as origin material according to a previous study (Tolkou et al. 2023, 2022). MgO (0.8 g), SiO_2_ (0.6 g), LaCl_3_ (1.8 g), or AlCl_3_ (0.8 g) were separately added to 5.0 g of activated carbon samples in 25 mL, aiming at its modification. Each content was mixed for 1 h at 298 K and sonicated for 2 h. After being filtered and washed with distilled water, they were dried over night at 333 K. These formed materials are further named as AC-Mg, AC-Si, AC-La, and AC-Al, respectively. Then, shares of these dried samples were mixed and calcined at 773 K for 5 h to obtain the AC-Mg-Si-La-Al (magnesium/silicate/lanthanum/aluminum) composite modified activated carbon and then cooled down at room temperature in order to be used in the experiments that follow.

### Analytical determinations

An ultraviolet–visible (UV–Vis) spectrophotometer (WTW Spectroflex 6100, Weilheim, Germany) was used for the determination of the residual concentration of RBBR at 593 nm (λ_max_) (Arya et al. 2020a), by corresponding the absorbance to the standard curve of RBBR.

### Characterization techniques

Scanning electron microscopy (SEM) (Jeol JSM-6390 LV, Japan scanning electron microscope)/EDS, Fourier transform infrared spectroscopy (FT-IR, Perkin Elmer, New York, NY, USA), and Brunauer, Emmett, and Teller (BET) analysis software, were used for the characterization of the surface of AC-Mg-Si-La-Al activated carbon.

### Adsorption experiments

For adsorption experiments, a specific amount of adsorbent has been introduced into Falcon tubes (15 mL) filled with appropriate concentrations of RBBR solution at constant temperature. Then, a Trayster overhead shaker and Loopster rotator were used for the agitation of the mixture at a specific agitation speed (80 rpm). pH value (3–9) of the solution, RBBR initial concentration (5–250 mg/L), dosage (0.1–0.5 g/L), and contact time (5–240 min) are some of the factors examined in the following experiments, as derived from preliminary experiments (results not shown in this study). A 0.45-μm pore size nylon filter was used for the filtration of the collected water samples for further analysis. Results show the average of three experiments performed. The percentage removal (% R) of RBBR was determined from Eq. 1:

1 $$\mathrm{R }\left(\mathrm{\%}\right)= \left(\frac{{\mathrm{C}}_{0}-{\mathrm{C}}_{\mathrm{f}}}{{\mathrm{C}}_{0}}\right)\times 100$$

where *C*_0_ = initial RBBR concentration (mg/L) and *C*_f_ = final RBBR concentration (mg/L).

The adsorption capacity of adsorbent (*Q*_e_) (mg/g) was calculated from Eq. 2:

2 $${\mathrm{Q}}_{e} = \frac{({\mathrm{C}}_{0}-{\mathrm{C}}_{\mathrm{e}})\times \mathrm{V}}{\mathrm{m}}$$

where *C*_e_ = RBBR concentration (mg/L) at equilibrium, *V* = volume of solution (L), and *m* = mass of the adsorbent used (g).

#### Isotherm models

A fixed amount of AC-Mg-Si-La-Al (g) was mixed with 10 mL of RBBR solution (5–250 mg/L). Even though there are many isotherm models (i.e., Dubinin-Radushkevich, Khan, Langmuir–Freundlich), in order to quantitatively evaluate the adsorption results, the most widely used models (Kalam et al. 2021), Langmuir (Eq. 3) and Freundlich (Eq. 4), were selected for fitting the equilibrium data.

3 $${\mathrm{Q}}_{\mathrm{e}}= \frac{{\mathrm{Q}}_{\mathrm{m}}{\mathrm{K}}_{\mathrm{L}}{\mathrm{C}}_{\mathrm{e}}}{1+{\mathrm{K}}_{\mathrm{L}}{\mathrm{C}}_{\mathrm{e}}}$$

4 $${\mathrm{Q}}_{\mathrm{e}} = {\mathrm{K}}_{\mathrm{F}}{\mathrm{C}}_{\mathrm{e}}^{1/\mathrm{n}}$$

where *Q*_m_ = theoretical monolayer/maximum adsorption capacity (mg/g), *K*_L_ = energy of RBBR adsorption (L/mg), *K*_F_ = constant related to adsorption capacity, 1/n = constant related to the intensity of adsorption or surface heterogeneity, and *C*_e_ = RBBR concentration (mg/L) at equilibrium.

The Langmuir model assumes monolayer coverage and characterizes chemisorption at specified adsorption sites, without interactions among adsorbed molecules. Conversely, Freundlich isotherm model assumes multilayer coverage as it is indicated for surface heterogeneity, indicating physisorption at the surface.

In this study, non-linear isotherm models were used, as it has been reported in the literature that non-linear modeling better represents the experimental results compared to linear ones (El-Khaiary and Malash 2011; Subramanyam and Das 2014).

#### Kinetics experiments

Pseudo-first-order (PFO) and pseudo-second-order (PSO) kinetic models of RBBR adsorption were chosen to fit the kinetic data of the experiments. The physical significance of this equation is their suitability for either long (PFO) (Eq. 5) or short (PSO) (Eq. 6) times of adsorption (Kyzas et al. 2012).

5 $${\mathrm Q}_{\mathrm t}={\mathrm Q}_{\mathrm e}(1-\mathrm e^{-{\mathrm k}_1\mathrm t})$$

6 $${\mathrm{Q}}_{\mathrm{t}}=\frac{{\mathrm{k}}_{2}{\mathrm{Q}}_{\mathrm{e}}^{2}\mathrm{t}}{1+{\mathrm{k}}_{2}{\mathrm{Q}}_{\mathrm{e}}\mathrm{t}}$$

where *Q*_t_ = RBBR adsorbed (mg/g) at time *t* (min) and *Q*_e_ = RBBR adsorbed (mg/g) at equilibrium. *k*_1_ = pseudo-first-order rate constant (L/min), *k*_2_ = pseudo-second-order rate constant adsorption (g/mg min), and *t* = contact time (min).

#### Thermodynamics

In this study, three significant thermodynamic parameters, the change of Gibbs free energy (*∆G*^*0*^*,* kJ/mol)*,* enthalpy (*∆H*^*0*^*,* kJ/mol), and entropy (*∆S*^*0*^, kJ/mol·K), are considered to estimate the adsorption procedure thermodynamically and determine the potential spontaneous nature. Therefore, to calculate the thermodynamic parameters, four different temperatures (298, 308, 318, and 338 K) were conducted and the following equations (Smith et al. 2018):

7 $${\mathrm{K}}_{\mathrm{c}}=\frac{{\mathrm{C}}_{\mathrm{s}}}{{\mathrm{C}}_{\mathrm{e}}}$$

8 $${\Delta G}^{0}= {-\mathrm{RTln}(\mathrm{K}}_{\mathrm{c}})$$

9 $${{\Delta G}^{0}=\Delta H}^{0}-\mathrm{T}{\Delta S}^{0}$$

10 $${\mathrm{ln}(\mathrm{K}}_{\mathrm{c}})=\left(-\frac{{\Delta H}^{0}}{\mathrm{R}}\right)+\frac{{\Delta S}^{0}}{\mathrm{R}}$$

*ΔG*^*0*^ was given from Eq. (8), and the values of *ΔH*^*0*^ and *ΔS*^*0*^ were calculated from the slope and intercept of the plot of ln(Kc) versus 1/T (Eq. (10)).

## Results and discussion

### Characterizations

#### Physical properties

**Fig. 2:**
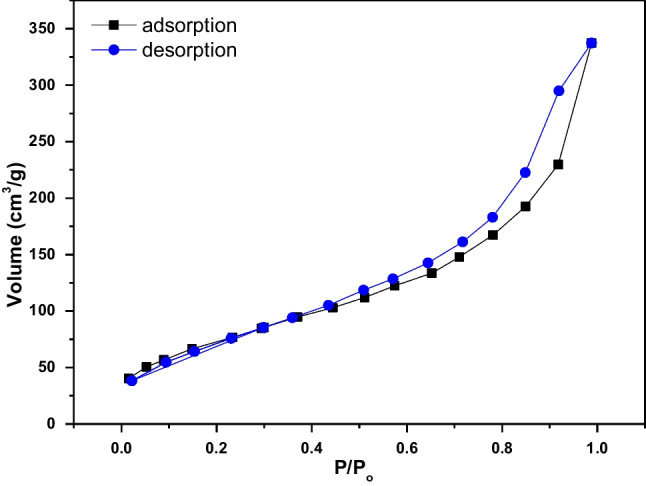
N_2_ adsorption–desorption isotherms of AC-Mg-Si-La-Al adsorbent versus relative pressure (P/P_o_) ranging from 0 to 1

According to N_2_ adsorption–desorption isotherms of AC-Mg-Si-La-Al adsorbent versus relative pressure (P/P_o_) shown in Fig. 2, AC-Mg-Si-La-Al displays type IV isotherms with hysteresis loops (Sotomayor et al. 2018), confirming the mesoporous structure of this adsorbent. The values of BET specific surface area, median Barrett-Joyner-Halenda (BJH) pore size and pore volume for AC-Mg-Si-La-Al are presented in Table 1. The co-presence of Mg, Si, La, and Al on AC tolerates simpler and quicker physisorption of N_2_; therefore, the curves overlap to the critical region. BET is then calculated by the desorption curve. The results showed that the AC-Mg-Si-La-Al displays a surface area of 289 m^2^/g, which is slightly lower than the corresponding values of other magnesium- and silica-modified activated carbons found in the literature (Mohamed et al. 2022).

**Table 1:** Physical properties of AC-Mg-Si-La-Al

Parameters	AC-Mg-Si-La-Al
BET Surface area, *S*_BET_ (m^2^/g)	289.32
Micropore volume, *V*_micro_ (cm^3^/g)	0.007
Total pore volume, *V*_T_ (cm^3^/g)	0.840

#### SEM/EDS

**Fig. 3:**
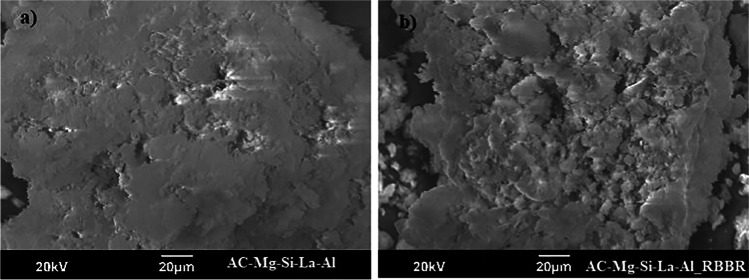
SEM images of AC-Mg-Si-La-Al adsorbent **a** before and **b** after RBBR adsorption

The SEM images of AC-Mg-Si-La-Al before and after RBBR adsorption are shown in Fig. 3. As illustrated in Fig. 3a, the surface of AC-Mg-Si-La-Al shows a rough texture spread over the surface. After RBBR adsorption (Fig. 3b), the surface of the adsorbent was converted to be more compact and smoother due to the filling of RBBR molecules on the AC-Mg-Si-La-Al surface. These results obtained from the SEM analysis identified that the morphology of AC-Mg-Si-La-Al is appropriate for RBBR dye adsorption.

**Fig. 4:**
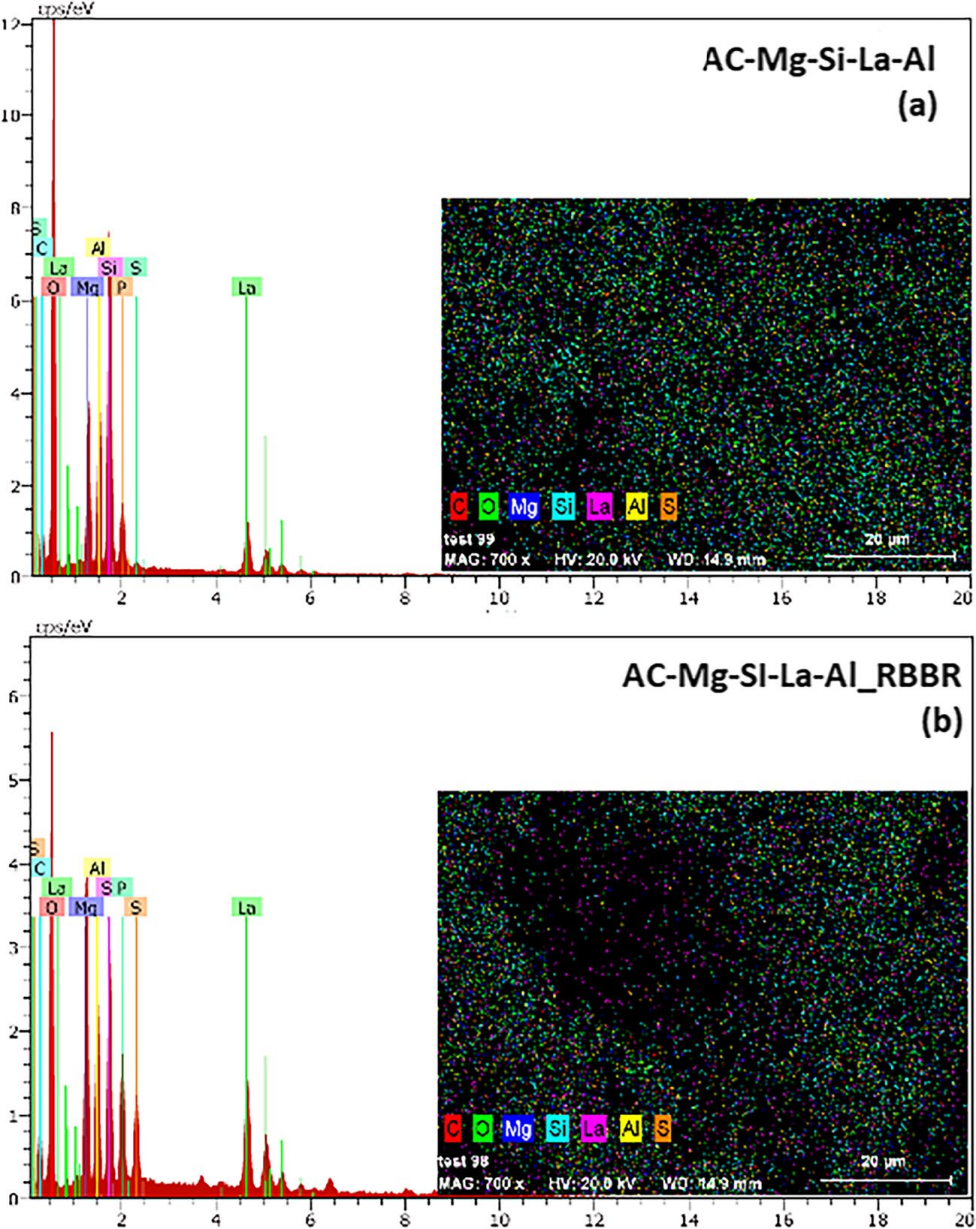
SEM/EDS analysis of AC-Mg-Si-La-Al adsorbent **a** before and **b** after RBBR adsorption

The SEM–EDS analysis of AC-Mg-Si-La-Al before and after RBBR adsorption is shown in Fig. 4, and the relative percentages of the elements found on the surface are presented in Table 2. As shown, Mg, Si, La, and Al were noticed on the surface of activated carbon, verifying carbon modification with a homogeneous distribution of these elements on the surface. Furthermore, after the adsorption of RBBR (Fig. 4b) is observed a greater percentage of carbon and sulfur in the surface of AC-Mg-Si-La-Al, which is possibly due to the additional carbon and sulfur that resulted from the structure of the adsorbed dye. In addition, after adsorption, a decrease in the percentage of silicon and aluminum is observed, possibly related to the binding of the dye as the presence of silicon was found to increase the surface affinity of the activated carbon with the dye (Mohamed et al. 2022).

**Table 2:** SEM/EDS analysis of AC-Mg-Si-La-Al adsorbent before and after RBBR adsorption

% (w/w)	AC-Mg-Si-La-Al	AC-Mg-Si-La-Al_RBBR
Lanthanum	1.35	1.68
Magnesium	5.05	5.66
Carbon	9.52	15.23
Silicon	6.51	2.47
Aluminum	4.08	2.67
Phosphorus	1.50	1.40
Sulfur	0.10	0.87
Oxygen	71.89	70.00

#### FTIR

**Fig. 5:**
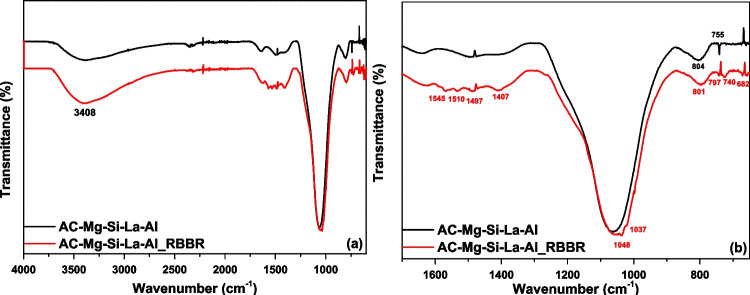
FTIR spectra of AC-Mg-Si-La-Al before and after RBBR adsorption (**a**) at 500-4000 cm^-1^ and (**b**) at a smaller scale of 600-1700 cm^-1^ for better visualization of the most characteristic peaks

The FTIR of AC-Mg-Si-La-Al-modified activated carbon before and after adsorption of RBBR is shown in Fig. 5. Observing the spectrum after adsorption, a broad peak at 3408 cm^−1^ (Fig. 4a) is shown which is limited in intensity to the spectrum of activated carbon before adsorption and is due to the water molecules (H_2_O) (symmetric and asymmetric O–H stretching vibration) that may have entered from the solvent (Arya et al. 2020a). Furthermore, the most characteristic peaks and variations are seen on a closer scale in Fig. 5. Thus, the bands at around 1545–1407 cm^−1^ do not protrude before adsorption but become apparent after adsorption and could attributed to the C = C stretching vibration in the aromatic rings which are for the anthraquinone dye RBBR (Arya et al. 2020a), confirming the adsorption of dye on the surface of AC-Mg-Si-La-Al. Moreover, the absorption band at 1547 cm^−1^ is attributed to the C = C stretching or amide-II (protein N–H bend, or C–N stretch) (Ozturk and Silah 2020) and at 1037 cm^−1^ is attributed to the S = O stretching vibration from sulfonic (–SO_3_^−^) groups (Sangar et al. 2019) that both exist at RBBR structure and is not appeared before adsorption. The intense broad peak seen at 110 cm^−1^, in the wide region 1000–1200 cm^−1^, both presented before and after the adsorption, corresponding to the Si − O − Si bond (Choong et al. 2020) and to the infrared vibration of C-O (Kazeem et al. 2018). Additionally, observing both spectra, a sharp peek at 1487 and 674 cm^−1^ can be assigned to MgO stretching vibrations (Selvam et al. 2011), while at 801–804 cm^–1^ is a suggestive peak for the stretching vibration of La = O groups (Aghazadeh et al. 2011).

### Batch adsorption experiments

#### Effect of adsorbents modification on dye adsorption

**Fig. 6:**
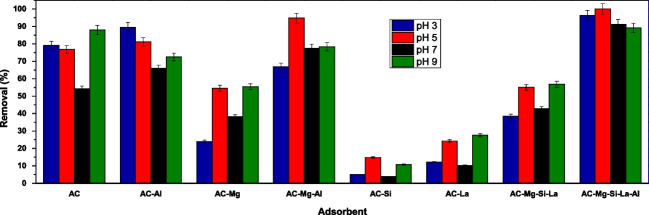
Comparison of adsorbents modification on RBBR removal; *C*_0_ 100 mg/L; pH 3.0, 5.0, 7.0, and 9.0 ± 0.1; dose 0.5 g/L; *T* = 298 K

In batch experiments, the effect of modification type of AC was examined to determine the efficiency of the adsorbents in RBBR removal. To test the effectiveness, an indicative dose of all applied adsorbents (0.5 g/L) was used at different pH values, to cover the acidic, neutral, and basic conditions, using 100 mg/L initial dye concentration at room temperature, and the results are shown in Fig. 6. As depicted, the effectiveness of AC in the adsorption of RBBR in some cases increases with its modification, and in some others a significant degradation is observed. In particular, the modification only with magnesium (AC-Mg), lanthanum (AC-La), and especially when modifying it with silicon (AC-Si) exhibits very low removal rates in all examined pH values. On the other hand, when aluminum participates in the modification of activated carbon (AC-Al, AC-Mg–Al and AC-Mg-Si-La-Al), its effectiveness increases relative to unmodified carbon. According to literature (Kazeem et al. 2018) the deposit of Al on the surface of AC enriches the pore structure for improved adsorption. Moreover, when all the additional components participate in the modification (AC-Mg-Si-La-Al), the effectiveness of the activated carbon increases impressively at all pH values, reaching the complete removal of RBBR dye (100%) at pH 5.0 ± 0.1. In addition, observing the removal rates of AC-Mg–Al material, at this pH value (95%), the combination of aluminum and magnesium is the one that contributes to this increase in efficiency, as magnesium may ionize the surface of AC to produce cationic and anionic molecules (Mg^2+−^OH^−^) that can enhance the adsorption of dyes (Ghalehkhondabi et al. 2021; Kittappa et al. 2020). According to recent literature, the composite with aluminum/magnesium showed preferential adsorption of anionic dyes (Grover et al. 2022).

In conclusion, this composite adsorbent material AC-Mg-Si-La-Al is chosen to be further studied in terms of its effectiveness and the possible mechanism that takes place for this complete dye removal.

#### Effect of adsorbent dose and initial pH on dye adsorption

**Fig. 7:**
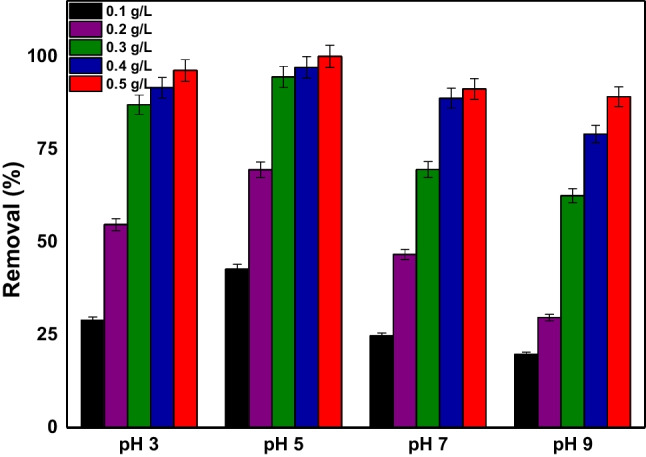
Effect of adsorbent dose and pH on RBBR removal; *C*_0_ 100 mg/L; pH 3.0, 5.0, 7.0, and 9.0 ± 0.1; dose 0.1–0.5 g/L; *T* = 298 K

The effect of the initial pH of the solution is an important factor that can affect the performance of adsorbents in water and wastewater treatment. Hence, the pH range 3.0 to 9.0 ± 0.1 at an initial dye concentration of 100 mg/L was studied and the relative results are shown in Fig. 7. In the same time, the effect of adsorbent’s dosage was studied, in order to define the optimum combination of dosage and pH for adsorption procedure. As depicted in Fig. 7, with increasing the dosage of AC-Mg-Si-La-Al, the removal efficiency of RBBR dye increases, as expected, at all considered pH values. Moreover, complete removal for RBBR dye (100%) was achieved by adding 0.5 g/L of adsorbent at acidic pH values. It is worth noting that at pH 5.0 ± 0.1, with the addition of just 0.2 g/L, 70% of the dye was removed and with 0.3 g/L the removal rate reached 95%.

In aquatic solutions, RBBR dye’s behavior is as follows (Mate and Mishra 2020):

$$\mathrm{X}-{\mathrm{SO}}_{3}\mathrm{Na}\rightleftarrows \mathrm{X}-{\mathrm{SO}}_{3}^{-}+{\mathrm{Na}}^{+}$$

**Fig. 8:**
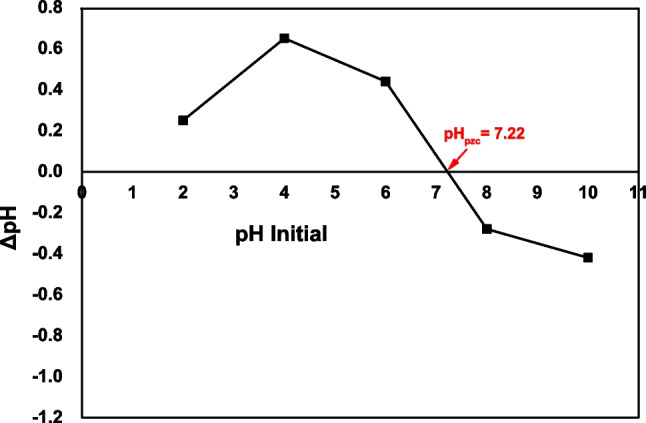
Determination of pH_pzc_ using pH drift method

The pH_pzc_ (point of zero charge) of AC-Mg-Si-La-Al adsorbent was measured in the range of 2–10 ± 0.1. pH_pzc_, which is the point at which the material’s surface charge becomes neutral, calculated by the relative curve plotted against ΔpH vs pH_initial_ using pH drift method. As shown in Fig. 8, the relative pH_pzc_ value was 7.22. For pH > 7.22, mainly negatively charged surface of AC-Mg-Si-La-Al is occurred (Kyzas et al. 2013). At pH < 7.22, the surface charge may get mostly positively charged. This meant that the positive surface charge of AC-Mg-Si-La-Al would be electrostatically attracted to the RBBR when pH is below 7.22. Therefore, at pH 5.0 ± 0.1, there is a full dye removal (100%), as at acidic pH, the dye molecule behaves as a cation due to protonation of the NH_2_ group (NH_3_^+^) (Arya et al. 2020a) and enhances its adsorption on the cation exchange sites. On the other hand, the negative surface charge would facilitate the interaction between adsorbent molecules and RBBR molecules (negatively charged form due to SO_3_^−^ groups) via electrostatic repulsion at pH higher than 7.22, due to competition between excess hydroxyl ions and negatively charged dye ions for adsorption binding sites (Parimelazhagan et al. 2022).

In basic conditions, carboxylate group present in the modified AC is converted into a carboxylate as follows (Mate and Mishra 2020):

$$-\mathrm{COOH}+\mathrm{NaOH}\rightleftarrows -\mathrm{COONa}+$$

These negatively charged carboxyl ions repelled the anionic dye resulting in a reduction in adsorption efficiency. Moreover, according to the literature (Ada et al. 2009; Aracagök, 2022; Arya et al. 2020b, 2020a; Kyzas et al. 2012; Ozturk and Silah 2020; Parimelazhagan et al. 2022), low pH value is usually ideal for the removal of RBBR dye, which is in agreement with the present study.

Finally, all adsorption studies that follow in this study performed at optimum pH 5.0 ± 0.1 and at a constant dosage of 0.4 g/L (97% removal).

### Effect of contact time

**Fig. 9:**
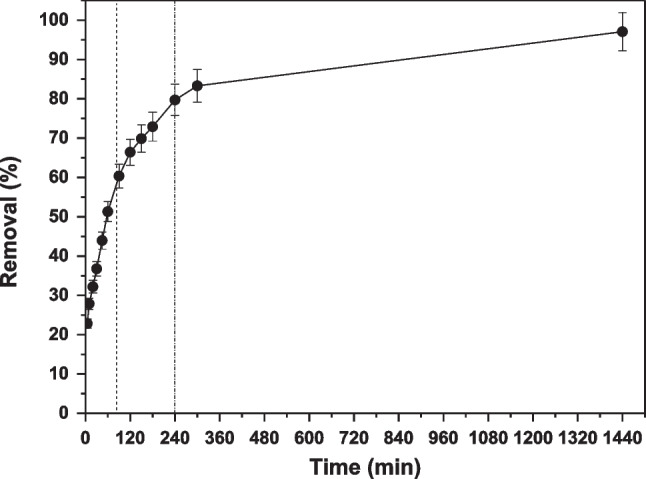
Effect of contact time on RBBR adsorption on AC-Mg-Si-La-Al; *C*_0_ 100 mg/L, pH 5.0 ± 0.1, dose 0.4 g/L, *T* = 298 K

In Fig. 9 is shown the effect of contact time on adsorption. Five to 1440 (24 h) min was the examined contact time range and was found that after 4 h (240 min) of reaction, additional increase in dye removal was insignificant, and thus, 4 h of reaction was chosen for further batch experiments. In particular, up to 80 min, there is an instantaneous dye adsorption (reaching 60% removal), indicating rapid external distribution and surface adsorption. Between 80 and 240 min, there is a slow equilibrium increasing removal rate to 80%, and after 300 min (5 h) up to 1440 min (24 h), an equilibrium state is achieved (Kyzas et al. 2012; Wang and Wang 2008). As it appears, the adsorption of RBBR is fast at the early stage of the contact time, where several sites on the surface are available for adsorption, but progressively is delayed until it reaches equilibrium, where the rest of the surface sites are harder to occupy, because of the existing repulsion among the solute molecules of the solid and the bulk phase.

### Adsorption isotherms

**Fig. 10:**
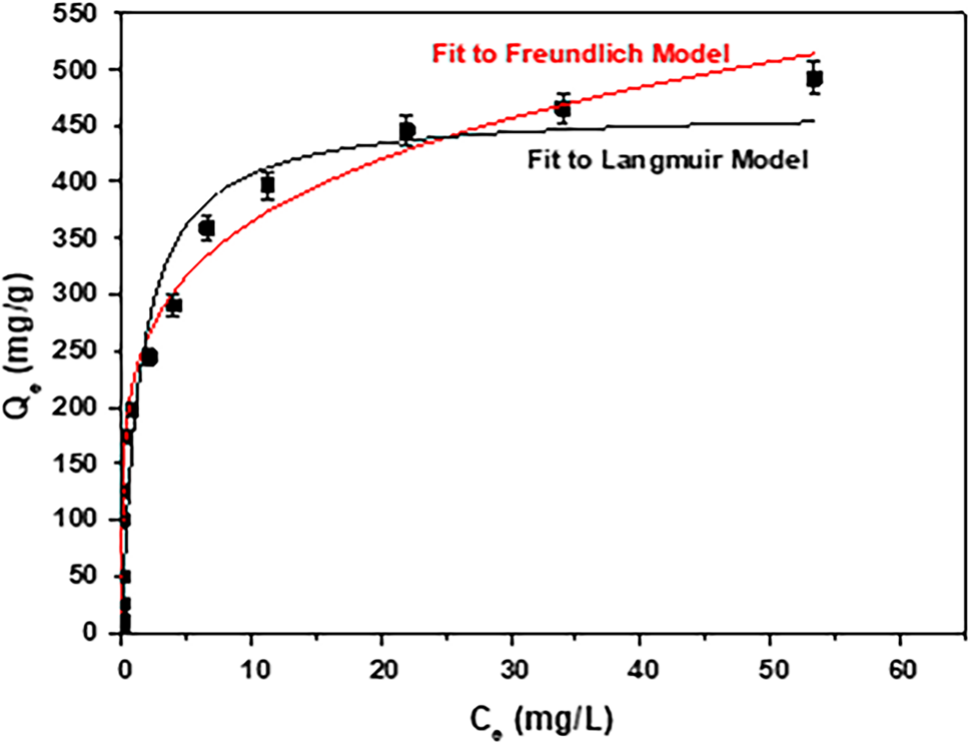
Freundlich and Langmuir isotherm models for the adsorption of RBBR (2–250 mg/L) on AC-Mg-Si-La-Al; pH 5.0 ± 0.1, dose 0.4 g/L, *T* = 298 K

Two different isotherm models (Freundlich and Langmuir) were used for the evaluation of the equilibrium data of adsorption. Figure 10 presents the adsorption isotherms of RBBR dye on AC-Mg-Si-La-Al, and Table 3 displays the several isotherm parameters. Based on the correlation coefficient (*R*^2^), the adsorption of RBBR dye is better described by the Freundlich isotherm model (*R*^2^ = 0.9189 for Freundlich and 0.8735 for Langmuir, respectively). Moreover, according to the Freundlich isotherm model, the meaning of the *n* value is when *n* < 1 the adsorption is a chemical procedure, *n* = 1 the adsorption is linear, and when *n* > 1 the adsorption is physical (Ozturk and Silah 2020). The relative *n* value of this study is 4.8939 and since 1/n is less than one (calculated as 0.2043), the adsorption of RBBR dye onto AC-Mg-Si-La-Al is favorable, confirming also that is a physical process (Rashid et al. 2018). As Freundlich adsorption isotherm model is valid for heterogeneous surfaces, this high *n* value shows a strong interface between adsorbent surface and RBBR based on the strong affinity of cationic exchange sites (Ozturk and Silah 2020) of AC-Mg-Si-La-Al with dye. This agrees with the conclusion obtained from Fig. 6 that the combination of aluminum and magnesium is the one that contributes to this increase in efficiency. Moreover, the higher the value of *K*_F_, the more effective the adsorption performance is.

**Table 3:** Constants of Freundlich and Langmuir isotherm models for the adsorption of RBBR (2–250 mg/L) on AC-Mg-Si-La-Al; pH 5.0 ± 0.1, dose 0.4 g/L, *T* = 298 K

Freundlich isotherm model				Langmuir isotherm model		
1/n	*n*	*K*_F_ (mg/g)(L/mg)^1/n^	*R*^2^	*Q*_m_ (mg/g)	*K*_L_ (L/mg)	*R*^2^
0.2043	4.8939	227.55	0.9189	465.0	0.6969	0.8735

On the other hand, the Langmuir isotherm model was used to estimate the maximum adsorption capacity (Q_max_) (Ozturk and Silah 2020) that describes the total capacity of the AC-Mg-Si-La-Al for the RBBR as 465 mg/g. In addition, the Langmuir constant (K_L_) indicates the extent of interaction between adsorbate and the surface but the small value 0.697 L/mg indicates a weak interaction (Tolkou et al. 2023).

Table 4 compares the synthesized adsorbent material of this study (AC-Mg-Si-La-Al) with some revealing materials published in the recent literature, comparing various parameters. As it can be shown, acidic conditions are confirmed to be optimal for RBBR dye removal, with pH values ranging 2.0–6.0. The relative results, presented in Table 4, showed that in most of the adsorption studies a higher adsorbent dose was used, leading to higher adsorption capacities, but none of the specific materials being compared achieves 100% of dye removal.

**Table 4:** Comparison of adsorption capacity of the proposed adsorbent in this study, with other adsorbents in literature

Adsorbent	C_0_ (mg/L)	Dosage (g/L)	pH_init_	Q_max_ (mg/g)	Removal (%)	Ref
ACTOL^1^	4165	2.0	6.0	170	81	(Arya et al. 2020b)
Amberlyst A21^2^	100	1.0	2.0	208	89	(Ozturk and Silah 2020)
DSAC^3^	100	0.1	2.0	357	95	(Ahmad et al. 2015)
WSBAC^4^	150	7.0	2.0	54	76	(Parimelazhagan et al. 2022)
Jh-g-AM-cl-B^5^	10	5.0	6.0	9.9	90	(Mate and Mishra 2020)
Yarrowia lipolytica^6^	150	1000	2.0	121	99	(Aracagök, 2022)
Cs–C^7^	100	0.1	6.0	540	90	(Nandanwar et al. 2022)
UPA*(θ)*^8^	800	10.0	4.0	123	96	(Xu et al. 2021)
AC-Mg-Si-La-Al^9^	100	0.5	5.0	465	100	Present study

^1^Activated charcoal of Thuja orientalis leaves; ^2^macroporous polystyrene resin; ^3^durian seed activated carbon; ^4^*Juglans nigra* shell biomass activated carbon; ^5^Jhingan hydrogel; ^6^*Yarrowia lipolytica* biomass; ^7^chitosan-activated carbon composite; ^8^ultraporous alumina (θ); ^9^magnesium/silicate/lanthanum/aluminum composite–modified activated derived from coconut shells

The novelty of this material (AC-Mg-Si-La-Al) examined in the present study is that an activated carbon, produced by coconut shells and further modified by using magnesium, silicate, lanthanum, and aluminum and applied to the removal of RBBR, is first mentioned in literature. Moreover, it is worth noting that the dose of AC-Mg-Si-La-Al used is very low compared to literature, as shown in Table 4, i.e., only 0.5 g/L to reach complete removal. Moreover, according to Fig. 7, by adding even 0.3 g/L, the percentage removal is still very high (95%). Relative removal rates found in the literature range between 76 and 95% using however higher doses. In addition, AC-Mg-Si-La-Al showed a relatively high adsorption capacity (465 mg/g) compared to other materials, suggesting that it is a potential and effective adsorbent material for dye removal.

### Adsorption kinetics

**Fig. 11:**
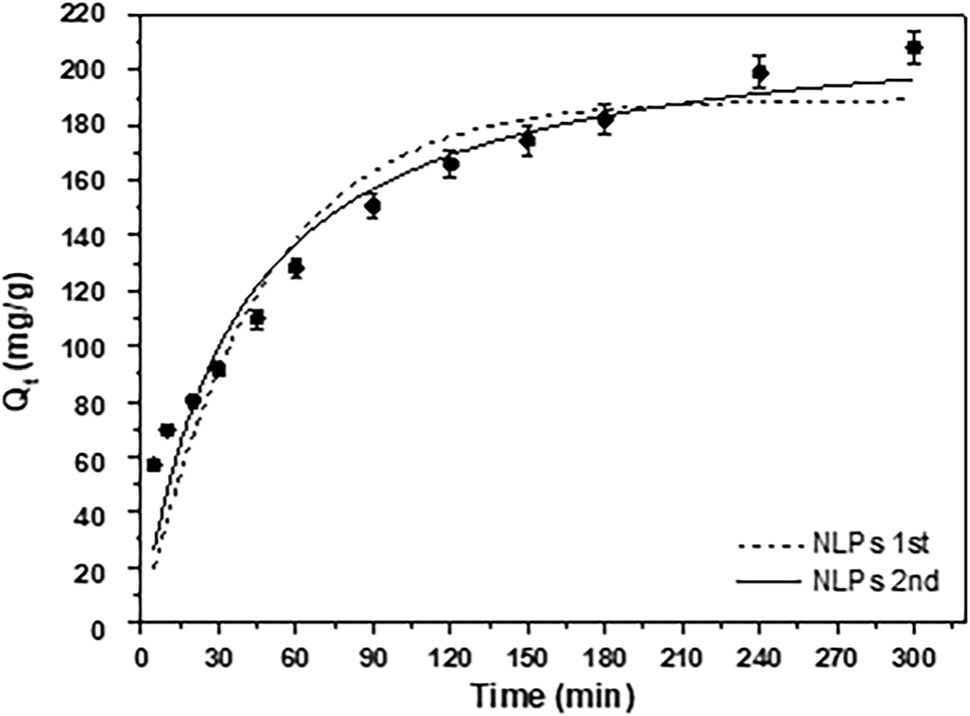
Kinetic pseudo-first- and pseudo-second-order model for the adsorption of RBBR on AC-Mg-Si-La-Al; C_0_ 100 mg/L, pH 5.0 ± 0.1, dose 0.4 g/L, *T* = 298 K

The adsorption of RBBR on AC-Mg-Si-La-Al was fitted to pseudo-first-order (PFO) and pseudo-second-order (PSO) models. These models and their non-linear forms are reported in Fig. 11 and Table 5. According to the results obtained, PSO model has the highest *R*^2^ value, thus 0.9291 in comparison with the relative low value of PFO (0.8695), indicating that that PSO model fits better to AC-Si-Mg-La-Al. In addition, the kinetic parameters showed that Q_e,cal_ (221 mg/g) calculated from Eq. (6) of pseudo-second-order model was similar to the experimental value (243 mg/g). Hence, adsorption kinetics study exhibited that the adsorption was closer to chemisorption instead of mass transfer which was about the exchange or sharing of electrons between dye and adsorbent (Arya et al. 2020a), and that was the determining step in the adsorption of RBBR.

**Table 5:** Pseudo-first and pseudo-second-order kinetic parameters for RBBR adsorption on AC-Mg-Si-La-Al

Q_e,exp_ (mg/g)	Pseudo-first order model			Pseudo-second order model		
	*K*_1_ (min^−1^)	*Q*_e,cal_ (mg/g)	*R*^2^	*K*_2_ (L/mg∙min)	*Q*_e,cal_ (mg/g)	*R*^2^
242.66	0.02199	189.52	0.8695	0.0124	220.83	0.9291

### Effect of ionic strength

**Fig. 12:**
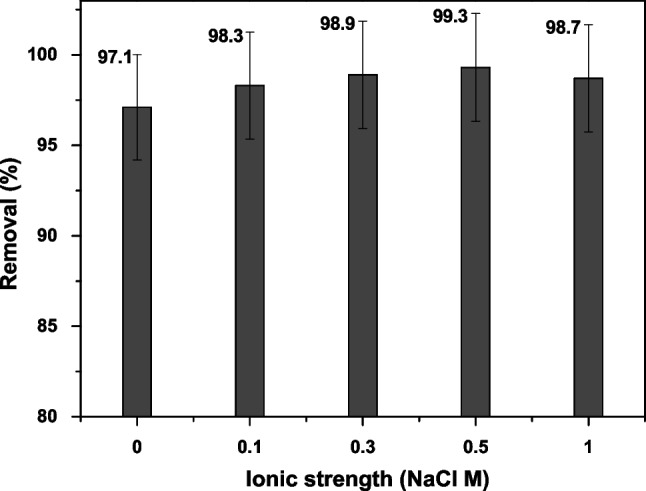
Effect of ionic strength on RBBR adsorption onto AC-Mg-Si-La-Al; C_0_ 100 mg/L, pH 5.0 ± 0.1, dose 0.4 g/L, T = 298 K, contact time 24 h

The effect of ionic strength on the adsorption of RBBR by adding a fixed amount of AC-Mg-Si-La-Al (0.4 g) was conducted by adding different concentrations of NaCl (0.1, 0.3, 0.5, and 1.0 M) during the adsorption experiment, following the same previous experimental procedure. According to the results shown in Fig. 12, a slight increase in the removal (%) of RBBR was observed, but with increasing NaCl concentration, a decrease in dye removal was seen. At 0.5 M ionic strength, the percentage of dye removed was 99.3% while at higher salt concentration (1.0 M), the relative percentage it only reached 98.7%, possibly because of the surface was less available to the dye removal at a greater salt presence (Khalaf et al. 2021). Therefore, sorption of RBBR became lower.

### Thermodynamics

The values of ΔH^0^ and ΔS^0^ were determined from the slop and intercept of the plot between ln(Kc) versus 1/T (*R*^2^ = 0.971, data is not displayed). The relative thermodynamic parameters, at all temperatures, are given in Table 6. As it results from the parameters, there is a positive value of ∆H^0^ (19.661 kJ/mol) that suggests the endothermic nature of the process (Kyzas et al. 2012). In addition, according to bibliography (Lima et al. 2019), the values of ∆G^0^ cannot be positive, as if the process of adsorption occurs, the corresponding values should be negative. That is because the negative values of ∆G^0^ recommend that the process of the anthraquinone acid dye adsorption is spontaneous (Kyzas et al. 2012; Liu 2009). Thus, adsorption is endothermic and the quantity of adsorbed molecules increases with increasing temperature. According to the positive value of ∆S^0^ (0.0737 kJ/mol∙K), there is an increase in random interaction between solid/liquid interfaces, which is because the water molecules, which are displaced by the dye molecules, gain more entropy than is lost.

**Table 6:** Thermodynamic parameters for the adsorption of RBBR on AC-Mg-Si-La-Al

T (K)	∆G^0^ (kJ/mol)	∆H^0^ (kJ/mol)	∆S^0^ (kJ/mol∙K)	*R*^2^
298	− 2.287	19.661	0.0737	0.971
308	− 3.023			
318	− 3.760			
338	− 5.233			

### Regeneration study

**Fig. 13:**
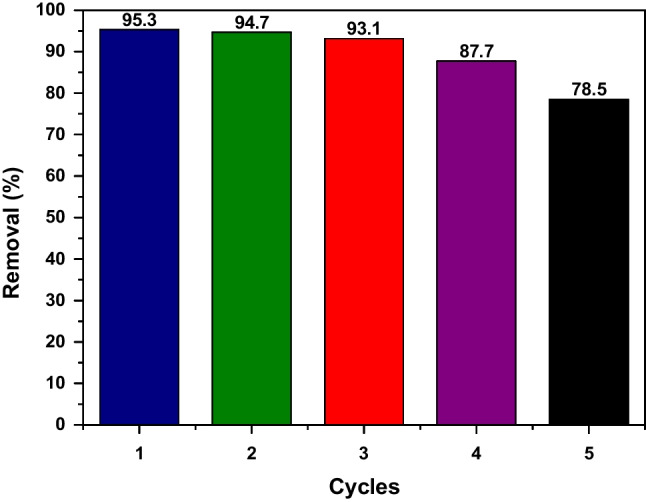
RBBR adsorption on AC-Mg-Si-La-Al; C_0_ 100 mg/L, pH 5.0 ± 0.1, dose 0.5 g/L, T = 298 K, contact time 3 h, for five adsorption–desorption cycles after regeneration at alkalic pH values, by using 1 M NaOH treatment

Regeneration experiments were applied to study the reusability of AC-Mg-Si-La-Al for the removal of RBBR dye (C_0_ 100 mg/L, pH 5.0 ± 0.1, dose 0.5 g/L) for five cycles. After the first cycle, the solids of AC-Mg-Si-La-Al were treated with 0.01 M NaOH and agitated for 3 h as the indicative repetitive time and then rinsed with distilled water for base removal. According to Fig. 13, at first cycle the removal (%) of RBBR dye was around 95% and after the fifth cycle it was reduced to 78%. Consequently, this work showed a reuse of AC-Mg-Si-La-Al adsorbent for five cycles of regeneration, showing only a 17% reduction in its efficiency, making it an effective and reusable material for many cycles.

## Conclusions

Activated carbon from coconut shells was used after modification with a mixture of metals and metalloids, such as magnesium, silicate, lanthanum, and aluminum (AC-Mg-Si-La-Al), for the removal of reactive anthraquinone dye RBBR. SEM, FTIR, and BET techniques were used for the characterization of the produced adsorbent. According to the results, the adsorbent has a surface area of 289 m^2^/g, has a rough texture spread over the surface, and has a homogeneous distribution of all elements on the surface confirming carbon impregnation, while FTIR results, before and after adsorption, confirmed the adsorption of RBBR on AC-Mg-Si-La, as specified by the peaks at 1547 cm^−1^ and 1037 cm^−1^ which are attributed to the amide-II (protein N–H bend, or C–N stretch) and to the S = O stretching vibration from sulfonic (–SO_3_^−^) groups, respectively.

Regarding the performance of the AC-Mg-Si-La-Al adsorbent, it was found that at pH 5.0 ± 0.1, with the addition of 0.3 g/L, the removal rate reached 95%, while a complete removal is achieved by adding 0.5 g/L. Moreover, at pH 5.0 ± 0.1, which is less than pH_pzc_ (7.22) of the adsorbent used, the positive surface charge of AC-Mg-Si-La-Al would be electrostatically attracted to the RBBR while at acidic pH, the dye molecule behaves as a cation due to protonation of the NH_2_ group and enhances its adsorption on the cation exchange sites.

The Freundlich isotherm model was found to better fit the adsorption (*R*^2^ = 0.92), than the application of Langmuir. Maximum Langmuir adsorption capacity was found to be 465 mg/g and 4 h (240 min) was selected as optimum time for the experiment. In addition, the results fitted better to the pseudo-second-order kinetic model, concluding that the adsorption of RBBR on AC-Mg-Si-La-Al was closer to chemisorption.

According to thermodynamics, there is a positive value of ∆H^0^ (19.661 kJ/mol) that suggests the endothermic nature of the process. Finally, a reuse of AC-Mg-Si-La-Al adsorbent for five cycles after successfully regenerated, showed only a 17% reduction in its efficiency, making it an effective and reusable material for many cycles.

The use of activated carbon derived from coconut shells co-modified with magnesium, silicate, lanthanum and aluminum was reported for the first time in this study for the removal of RBBR dye. Due to its efficiency in complete removal of RBBR, AC-Mg-Si-La-Al could be further used to remove many other dyes, even anionic or cationic ones.

## Data Availability

The datasets generated during and/or analyzed during the current study are available from the corresponding author on reasonable request.
